# Prognostic value of matrix metalloproteinases in oral squamous cell carcinoma

**DOI:** 10.1080/13102818.2014.967510

**Published:** 2014-11-29

**Authors:** Georgi Mishev, Elitsa Deliverska, Ruslan Hlushchuk, Nikolay Velinov, Daniel Aebersold, Felix Weinstein, Valentin Djonov

**Affiliations:** ^a^ Institute of Anatomy, University of Bern, Bern, Switzerland; ^b^ Department of Oral and Maxillofacial Surgery, Faculty of Dental Medicine, Medical University of Sofia, Sofia, Bulgaria; ^c^ Department of Radiation Oncology, Inselspital, Bern University Hospital, Bern, Switzerland

**Keywords:** matrix metalloproteinase, oropharyngeal squamous cell carcinoma, tumour progression

## Abstract

The aim of this study was to investigate whether there is a correlation between the expressions of four matrix metalloproteinases (MMPs): MMP-2, MMP-7, MMP-9 and MMP-13, and the TNM (tumour–node–metastasis) stages of oral squamous cell carcinoma (OSCC); and to explore the implication of these MMPs in OSCC dissemination. Samples from 61 patients diagnosed with oropharyngeal tumour were studied by immunohistochemistry against MMP-2, MMP-7, MMP-9 and MMP-13. The assessment of immunoreactivity was semi-quantitative. The results showed that MMP-2 and MMP-9 had similar expression patterns in the tumour cells with no changes in the immunoreactivity during tumour progression. MMP-9 always had the highest expression, whereas that of MMP-2 was moderate. MMP-7 showed a significant decrease in expression levels during tumour evolution. MMP-13 had constant expression levels within stage T2 and T3, but showed a remarkable decline in immunoreactivity in stage T4. No significant differences in the MMPs immunoreactivity between tumour cells and stroma were observed. Although strong evidence for the application of MMPs as reliable predictive markers for node metastasis was not acquired, we believe that combining patients’ MMPs expression intensity and clinical features may improve the diagnosis and prognosis. Strong evidence for the application of MMPs as reliable predictive markers for node metastasis was not acquired. Application of MMPs as prognostic indicators for the malignancy potential of OSCC might be considered in every case of tumour examination. We believe that combining patients’ MMPs expression intensity and clinical features may improve the process of making diagnosis and prognosis.

## Introduction

Squamous-cell carcinoma (SCC) is a highly aggressive and malignant form of tumour, with a very poor prognosis. At least 90% of all oral malignancies are of this kind. In the world-incidence ranking of cancers, it holds the 8th position.[1] oral squamous cell carcinoma (OSCC) can invade the adjacent tissue and spreads fairly rapidly. It has a notorious tendency to invade the regional lymph nodes, probably at a very early stage of tumourogenesis. Consequently, well-nigh 50% of the patients who undergo therapy develop distant metastases, which binds them to an uninterrupted course of treatment until death intervenes.[2–5] The localization of the primary tumour plays a crucial role in its dissemination, close anatomic relationships with lymphatic and blood vessels being a prerequisite for early metastases.

The metastatic potential of OSCC depends upon its ability to digest the extracellular matrix (ECM), to penetrate the basement membrane (BM), to initiate tumour angiogenesis,[6–9] and to invade the adjacent tissues and vessels. The BM, which separates the epithelium from the mesenchymal tissue, is the first barrier against invasion. Degradation of the ECM and the BM requires the participation of matrix metalloproteinases (MMPs). Most of these enzymes are involved in common physiological processes, such as the proliferation, differentiation and apoptosis of cells, angiogenesis, and the morphogenesis and repair of bodily tissues.[10–16] In the early 1980s, Liotta et al. [17] identified proteolysis, and, specifically, the digestion of type-IV collagen, to be essential for the invasiveness of melanomas. Formerly, MMPs were deemed to be expressed exclusively within tumour cells, but this belief has since been refuted. Their up-regulation therein is now known to be triggered by the host's stromal cells.[18,19] It has been postulated that the invasion potential of OSCCs is conferred by their ability to utilize MMPs that are produced by the host's stromal cells.[20–22] Yorioka et al. [23] have suggested that tumour cells either stimulate the liberation of MMPs from the host's stromal cells, thereby favouring the proteolytic degradation of the ECM or themselves synthesize these proteinases. Although some authors argue that the zymographic detection of the gelatinolytic activity of MMPs is more representative than their immunohistochemical mapping, Ikebe et al. [24] have demonstrated the existence of a significant correlation between the results obtained using these two methods.

To date, 25 members of the MMP family have been identified. They are classified according to their amino-acid-sequence homology either as soluble enzymes (collagenases, gelatinases, stromelysins and an elastinase), or as membrane-associated ones.[15] Most of the MMPs are secreted in an inactive form, which is activated pericellularly or extracellularly by serine proteases such as trypsin, plasmin or neutrophil elastase. MMPs are down-regulated by endogenous tissue inhibitors of metalloproteinases (TIMPs).[11,25] The role of MMPs in tumourigenesis and angiogenesis has been confirmed by many authors during the past few decades. The latter process is essential for the delivery of nutrients to the proliferating cancer cells and for furnishing the structural support for tumour expansion.[26,27] MMPs do not act synergically during tumourigenesis: the up-regulation of MMP-2 and MMP-9 is associated with the degradation of the ECM and the BM, and with an increase in tumour aggressiveness; the down-regulation of MMP-13 is associated with a worsening of the disease prognosis; and that of MMP-7, with an increase in tumour invasiveness.

MMPs have also been demonstrated to be involved in the progression of tumours and in their metastatic dissemination. Given the important role played by this family of enzymes in degrading the BM and the ECM, their participation in the propagation of tumour cells is perhaps not surprising. Selected MMPs have been implicated in angiogenesis, a process without which a tumour cannot undergo the neovascularization that is crucial for its nourishment and structural support.[3,26,28–31] Certain members of the MMP family are the only proteinases that are known to be capable of cleaving the collagen types of which the BM (type IV) and the ECM (types I and III) are composed,[32] MMP-2 and MMP-9 degrade type-IV [21,33,34] and MMP-13, types I to IV [35]. MMP-7 degrades fibronectin, tenascin and β4 integrin, which play a crucial role in the adhesion and migration of cells during tumourigenesis.[12,30,31,36,37]

The aim of this study was to define the role played by MMP-2, MMP-7, MMP-9 and MMP-13 in the progression and invasion of OSCC and to ascertain whether they could serve as reliable markers of these phenomena. To this end, we mapped immunohistochemically their expression profiles within tumour samples that were derived from 61 patients with OSCC, and correlated these data with histological findings that were classified according to Bryne's malignancy-grading system. The distribution of MMPs within OSCCs was mapped immunohistochemically because this technique (1) permits a direct correlation with morphological data, and (2) can be performed on paraffin-embedded tissue sections that are routinely produced for diagnostic purposes.[38] Hence, the technique could be performed on a regular basis even in hospitals.

## Materials and methods

This study was approved by the Regional Board of Medical Ethics.

Tumour samples were derived from 61 patients who had been diagnosed as suffering from OSCC and who had been treated at Inselspital in Bern. Paraffin-embedded samples were made available to us by the Department of Pathology. Information about clinicopathological covariables was gleaned from the medical records of the patients. The patients were administered a median total dose of 74 Gy (range: 54–80.5 Gy), which was delivered in daily fractions, five times a week for five to eight weeks. Ninety-seven per cent of the patients were exposed to at least 66 Gy.[39] The primary tumour and lymph-node metastases were evaluated separately. After treatment, all patients were clinically examined and imaged on a regular basis. Fifty-five patients were observed until the time of death, the median follow-up period being 2.6 years.

Of the 61 patients, 44 were male and 17 female. Their age ranged from 41 to 85 years. Fifty-two of the patients smoked at least one cigarette per day, and 52 drank an alcoholic beverage at least once a week (Table 1).

**Table 1.** Clinical data about the patients and the tumours.

Patients’ details	Number of patients
Age (41–85)	
Gender	
Male	44 (72%)
Female	17 (28%)
T classification	
2	6 (10%)
3	16 (26%)
4	39 (64%)
Tumour grade	
I	5 (8%)
II	42 (69%)
III	14 (23%)
N classification	
N0	16 (26%)
N1	8 (13%)
N2	29 (48%)
N3	8 (26%)
Distant metastasis	
Yes	50 (82%)
No	11 (18%)
Smoking habits	
Smoker	52 (85%)
Non-smoker	9 (15%)
Alcohol-consumption habits	
Drinker	52 (85%)
Non-drinker	9 (15%)

The patients were classified according to the Union for International Cancer Control TNM system (UICC-TNM) of 1997. Clinicopathologically, the tumours were graded on a scale from I to IV. According to the TNM system, 55 of the patients were at stage T3/4. In 45 of the patients, at least one lymph node was affected and 50 manifested distant metastasis. In 47 of the patients, the tumour was graded histologically as I, II, and in the remaining 14, as III.

### Immunohistochemistry

Three-micrometre-thick sections of the paraffin-embedded tumour samples were transferred to gelatinized micro-slides and air-dried overnight at 37 °C. They were deparaffinized in xylene (three changes of medium) and rehydrated in ethanol (100% through 30%). They were then washed three times in distilled water and twice in Tris-buffered saline (TBS) [50 mmol/L Tris/HCL (pH 7.4), containing 100 mmol/L sodium chloride]. To block non-specific binding, the sections were incubated in TBS containing 1% casein (SIGMA 8654) for 10 min. They were then exposed to primary antibodies against MMP-2, MMP-7, MMP-9, MMP-13, α-smooth-muscle actin (anti-α-SMA) (A-2547, Sigma-Aldrich-Chemie, Gmbh), diluted 1:200 in TBS, and rabbit anti-fibronectin (F 3648 Sigma-Aldrich-Chemie, Gmbh), diluted 1:200 in TBS. The latter two antibodies were applied to identify stromal-muscle cells and fibroblasts, respectively.

### Primary antibodies

The following primary antibodies were used: mouse anti-MMP-2 (Ab-4) VC2 (Neo Markers, Cat# MS-806-R7, Lot# 806R107, ready to use); rabbit polyclonal anti-MMP-7 (Ab-4) (Oncogene™, Cat# PC492, Lot# D18417-1) at a 1:100 dilution in Antibody-Diluent; mouse anti-MMP 7 Ab-1 (ID2) (NeoMarkers, Cat# MS-813-R7, Lot# 813R111, ready to use); rabbit anti-MMP-9 (NeoMarkers, Cat# RB-1539-R7, Lot# 1539R303, ready to use, ON –4 °C); mouse anti-MMP-13 Ab-2 (ID3) (Oncogene™, Cat# MS-826-R7, Lot# 826R909; ready to use, ON –4 °C).

The sections were then rinsed three times in TBS before incubating with the secondary antibodies for 45 min at ambient temperature.

### Secondary antibodies

The following secondary antibodies were used: Biotin-anti-Mouse Ig (DAKO EO 433) at a 1:200 dilution in TBS; Biotin-anti-Rabbit Ig (DAKO EO 353) at a 1:200 dilution in Antibody-Diluent; Biotin-anti-Rabbit Ig (DAKO EO 353) at a 1:200 dilution in TBS; Biotin-anti-Mouse Ig (DAKO EO 433) at a 1:200 dilution in TBS.

They were again rinsed three times in TBS and then treated with horseradish-peroxidase–streptavidin complex (P355, DAKO) likewise for 45 min at ambient temperature. The reaction product was visualized by exposing the slides to 3-amino-9-ethylcarbazole (Sigma). The specimens were then mounted in Aquatext (Merck). Tissue samples that were incubated with non-immune serum served as negative controls.

### Semi-quantitative evaluation of immunoreactivity for MMPs

Immunoreactivity for MMPs in each tumour sample was assessed in a blinded fashion and independently by two persons who had no prior knowledge of the clinical features. The intensity of the immunostaining reaction and the percentage of positively stained cells within the entire section area were graded as follows: (0): negative (no immunoreactivity); (1): minimal staining (<10% of the section area); (2): moderate staining (10%–50% of the section area); (3): strong staining (>50% of the section area).

For convenient description of the results, the intensity of the immunostaining reaction was subdivided into two broad categories: weak (grades 1 and 2) and strong (grades 3 and 4). Immunoreactivity was statistically correlated with clinical parameters, using Fisher's exact test.

The invasion front was evaluated histologically according to Bryne's malignancy grading system,[40] which yields a better prognostic value for SCCs [41] than the conventional one. Four morphological parameters were individually evaluated at the invasion front: (i) degree of keratinization; (ii) tumour structure (pattern of invasion); (iii) nuclear polymorphism; and (iv) the host's cellular inflammatory response. For each parameter, an average of four fields were evaluated under a light microscope at a final magnification of ×100.

Each parameter (i–iv) was graded on a scale from 1 to 4. Degree of keratinization: (1) highly keratinized (>50% of the cells); (2) moderately keratinized (20%–50% of the cells); (3) minimally keratinized (5%–20% of the cells); (4) no keratinization (0%–5% of the cells). Tumour structure (pattern of invasion): (1) advancing, well-delineated borders (solid sheets); (2) infiltrating solid cords, bands and/or strands; (3) small groups or cords of infiltrating cells (*n* < 15); (4) marked and widespread dissociation into small groups of cells and/or into single cells (*n* >15). Nuclear polymorphism: (1) minimal (mature cells: >75%); (2) moderate (mature cells: 50%–75%); (3) abundant (mature cells: 25%–50%); and (4) extreme (mature cells: 0%–25%). Host's cellular inflammatory response: (1) marked; (2) moderate; (3) slight; (4) none.

### Quantitative evaluation of immunoreactivity for MMPs

To quantify the expression pattern of MMPs at the invasive front, tissue immunoreactivity (Tiss) and the relative number of immunopositive cells (Rn) were computed within the 284 investigated fields. Initially, the number of fields with the same numerical combination of Tiss and Rn was calculated. Then, the number of different score combinations for Tiss and Rn (according to Bryne's grading system) was recorded.

## Results and discussion

The expression patterns of MMP-2, MMP-7, MMP-9 and MMP-13 in the tumour samples, both within the tumour cells (intratumoural) and within the intervening and surrounding stroma, were mapped immunohistochemically. Immunoreactivity was graded semi-quantitatively, and the data were correlated with histological findings that were classified according to Bryne's malignancy-grading system, as well as with clinicopathological features, namely the stage of the tumour and lymph-node involvement.

### MMPs expression patterns

Our results showed that in approximately 90% of the tumour samples (from 61 patients with OSCC), the immunostaining for MMP-2, MMP-7 and MMP-9 was classified as strong and these findings accord with those published by other authors.[38,42] Nevertheless, the intensity distributions of intratumoural and stromal staining were likewise comparable.

## MMP-2

**Figure 1.**
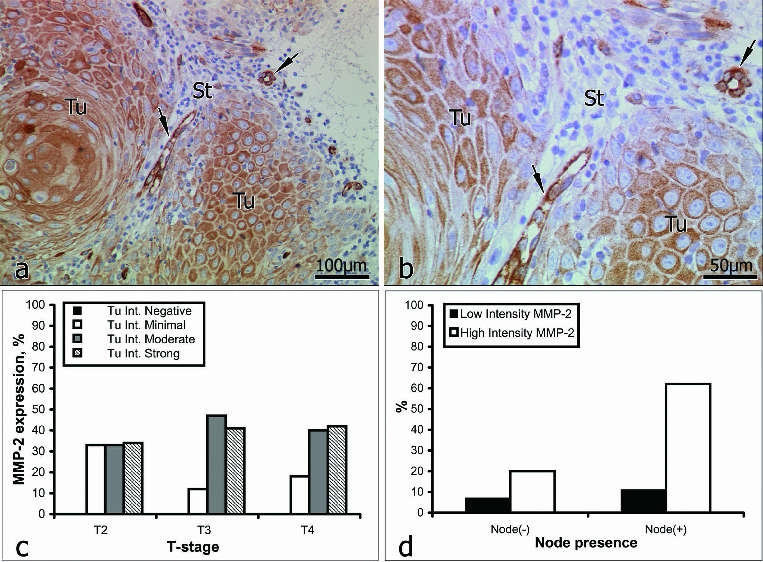
Immunoreactivity for MMP-2 (a and b): immunopositive vessels (*arrows*); St = stroma; Tu = tumour. Correlation between MMP-2 expression intensity and tumour stage (c); lymph-node involvement (d).

Intratumoural staining for MMP-2 (Figure 1) was graded as weak in 18% of the samples (11/61) and as strong in 82% (50/61). Within the intervening and surrounding stroma (Figure 1), immunostaining for MMP-2 was weak in 23% of the samples (14/61) and strong in 77% (47/61).

In patients *with no* lymph-node involvement (16/61), inratumoural staining for MMP-2 was weak in 25% of the cases (4/16) and strong in the other 75% (12/16). In patients *with* lymph-node involvement (45/61), intratumoural staining for MMP-2 was weak in 16% of the cases (7/45) and strong in the other 84% (38/45) (Table 2).

**Table 2.** Intensity of distribution of Tu and stromal staining for MMP-2.

MMP-2	Intensity of Tu staining				Intensity of stromal staining			
Intensity grade	Weak		Strong		Weak		Strong	
Tu stage	0	1	2	3	0	1	2	3
Tu2	0	2	2	2	0	1	3	2
Tu3	0	2	8	7	0	3	8	6
Tu4	0	7	15	16	0	10	17	11
Total	11 (18%)		50 (82%)		14 (23%)		47 (77%)	
Node (−)neg.	0	4	7	5	0	4	8	4
Total	4 (7%)		12 (20%)		4 (7%)		12 (20%)	
Node (+)pos.	0	7	18	20	0	10	20	15
Total	7 (11%)		38 (62%)		10 (16%)		35 (57%)	

Note: Tu – Tumour.

Node (−) neg. – Lymph-node involvement.

Node (+)pos. – Lymph-node involvement.

Although Kato et al. [20] propose the use of MMP-2 as a predictive marker for tumour progression and the early invasion of lymph nodes, according to our immunostaining data, the expression of MMP-2 was not closely associated with the stage of the tumour (Figure 1). In 62% of our patients with lymph-node involvement, immunostaining for MMP-2 was strong, but in 20% of those with no lymph-node involvement, immunostaining for MMP-2 was likewise strong (Figure 1, Table 2). This suggests that in patients with no lymph-node involvement at the time of treatment, the process of dissemination had most probably already begun. Therefore, on the basis of our data, we do not consider MMP-2 to be a reliable predictive marker of tumour invasiveness in OSCC, but other authors have reported the existence of a positive correlation between the expression of MMP-2, lymph-node recurrence and a worsening of the survival rate.[43–48]

### MMP-7

**Figure 2.**
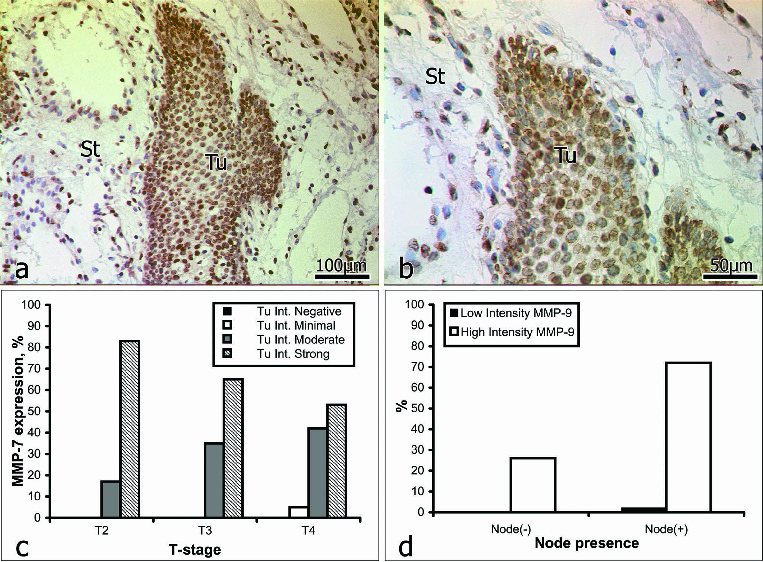
Immunoreactivity for MMP-7 (a and b): St = stroma; Tu = tumour. Correlation between MMP-7 expression intensity and tumour stage (c); lymph-node involvement (d).

Intratumoural staining for MMP-7 (Figure 2) was graded as weak in 3% of the samples (2/16) and as strong in 97% (59/61). Within the intervening and surrounding stroma (Figure 2), immunostaining for MMP-7 was weak in 8% of the samples (5/61) and strong in 92% (56/61). In patients *with no* lymph-node involvement (16/61), intratumoural staining for MMP-7 was weak in 6% of the cases (1/16) and strong in the other 94% (15/16). In patients *with* lymph-node involvement (45/61), intratumoural staining for MMP-7 was weak in 2% of the cases (1/45) and strong in the other 98% (44/45) (Table 3).

**Table 3.** Irrespective of the intensity distribution of stromal staining for MMP-7 corresponded to the intratumoural pattern.

MMP-7	Intensity of Tu staining				Intensity of stromal staining			
Intensity grade	Weak		Strong		Weak		Strong	
Tu stage	0	1	2	3	0	1	2	3
Tu2	0	0	1	5	0	0	4	2
Tu3	0	0	6	11	0	3	13	1
Tu4	0	2	16	20	0	2	29	7
Total	2 (2%)		59 (98%)		5 (8%)		56 (92%)	
Node(–)neg.	0	1	5	10	0	3	12	1
Total	1 (2%)		15 (24%)		3 (5%)		13 (21%)	
Node(+)pos.	0	1	18	26	0	2	34	9
Total	1 (2%)		44 (72%)		2 (3%)		43 (71%)

These results show the immunostaining for MMP-7 to be closely correlated with the stage of the tumour: strong staining decreased, and moderate staining increased with an advance in the stage of the tumour. That is, overall, the expression of MMP-7 declined with the progression of OSCC. There was a correlation between the expression of MMP-7 and lymph-node involvement. In 72% of the patients with lymph-node involvement, immunostaining for MMP-7 was strong, and likewise, immunostaining for MMP-7 was also strong in 24% of those with no lymph-node involvement (Figure 2, Table 3). These data do not fully accord with published findings; for example, Impola et al. [9] observed no correlation between immunoreactivity for MPP-7 and the stage of OSCC. However, the same authors have since reported the existence of a link between the expression of MMP-7, an inhibition of angiogenesis and a retardation of tumour growth.[8,49] Mimori et al. [37] have suggested that MMP-7 is involved either directly in tumour growth, or, indirectly, by activating the epidermal growth factor. In the light of this suggestion our data are interesting, as they indicate that MMP-7 could possibly serve as a predictive marker of tumour invasiveness in OSCC. Further studies on a larger cohort would, of course, be needed to prove this correlation.

### MMP-9

**Figure 3.**
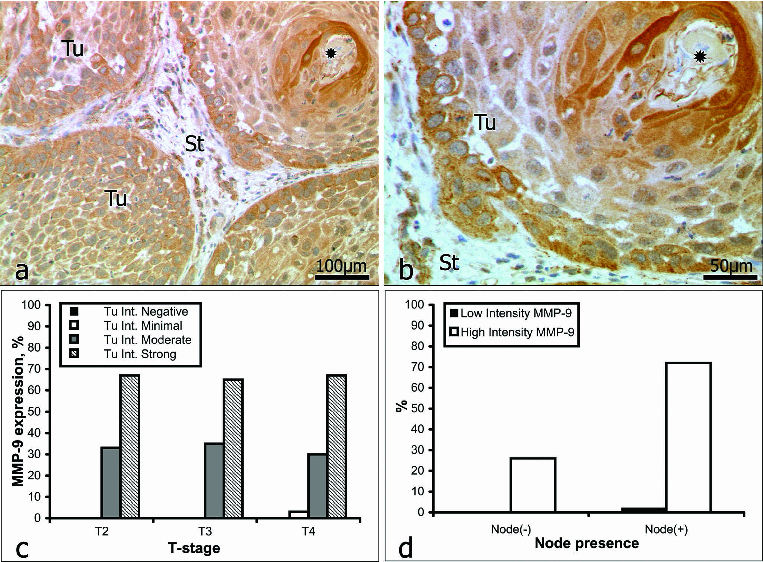
Immunoreactivity for MMP-9 (a and b): keratinized pearl (*asterisk*); St = stroma; Tu = tumour. Correlation between MMP-9 expression intensity and tumour stage (c); lymph-node involvement (d).

Intratumoural staining for MMP-9 (Figure 3) was graded as weak in 2% of the samples (1/61) and as strong in 98% (60/61). Within the intervening and surrounding stroma (Figure 3), immunostaining for MMP-9 was weak in 16% of the samples (10/61) and strong in 84% (51/61). In patients *with no* lymph-node involvement (16/61), intratumoural staining for MMP-9 was strong in all cases (16/16). In patients *with* lymph-node involvement (45/61), intratumoural staining was weak in 2% of the cases (1/45) and strong in the other 98% (44/45) (Figure 3, Table 4).

**Table 4.** Irrespective of lymph-node involvement, the intensity distribution of stromal staining for MMP-9 corresponded to the intratumoural pattern.

MMP-9	Intensity of Tu staining				Intensity of stromal staining			
Intensity grade	Weak		Strong		Weak		Strong	
Tu stage	0	1	2	3	0	1	2	3
Tu2	0	0	2	4	0	2	2	2
Tu3	0	0	6	11	0	0	14	3
Tu4	0	1	11	26	2	6	28	2
Total	1 (2%)		60 (98%)		10 (16%)		51 (84%)	
Node(−) neg.	0	0	7	9	0	3	11	2
Total	0 (0%)		16 (26%)		3 (5%)		13 (21%)	
Node(+) pos.	0	1	12	32	2	5	33	5
Total	1 (2%)		44 (72%)		7 (11%)		38 (63%)

MMP-9 has been detected in the plasma of patients with carcinomas and has been proposed as a good marker of head and neck SCC.[16] Our data, however, revealed no evidence of a correlation between immunostaining for MMP-9 and the stage of the tumour (Figure 3). Indeed, the incidence of both strong and moderate staining remained remarkably constant with the progression of OSCC. Katayama et al.[50] have reported the expression of MMP-9 to be significantly higher in patients with regional lymph-node or distant metastasis than in those without such. Our data that in 68% of the patients with lymph-node involvement immunostaining for MMP-9 was strong (Figure 3, Table 4) support these findings; but there are also reports that question the reliability of MMP-9 as a marker of head and neck SCCs.[34]

### MMP-13

**Figure 4.**
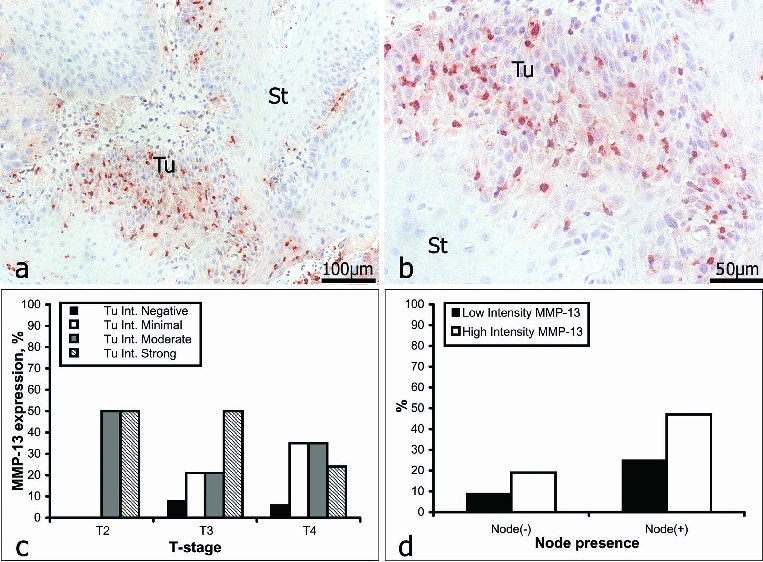
Immunoreactivity for MMP-13 (a and b): St = stroma; Tu = tumour. Correlation between MMP-13 expression intensity and tumour stage (c); lymph-node involvement (d). N.B.: For the analyses of MMP-13 a sufficiency of tissue was available from only 57 of the 61 patients.

MMP-13 is a potent collagenase, which is rarely expressed in normal tissue, but which is often up-regulated when a rapid turnover of the ECM is required, as during the local invasion and growth of a malignant tumour.[34,51] MMP-13 is expressed at high levels in SCCs of the head and neck.[52–54] In our study, the intratumoural staining for MMP-13 (Figure 4) was graded as weak in 33% of the samples (19/57) and as strong in 67% (38/57). Within the intervening and surrounding stroma (Figure 4), immunostaining for MMP-13 was weak in 39% of the samples (22/57) and strong in 61% (35/57). In patients *with no* lymph-node involvement (16/57), intratumoural staining for MMP-13 was weak in 31% of the analysed negative samples (5/16) and strong in the other 69% (11/16). In patients *with* lymph-node involvement (41/57), intratumoural staining for MMP-13 was weak in 34% of the analysed positive samples (14/41) and strong in the other 66% (27/41) (Figure 4, Table 5).

**Table 5.** Irrespective of lymph-node involvement, the intensity distribution of stromal staining for MMP-13 corresponded to the intratumoural pattern.

MMP-13	Intensity of Tu staining				Intensity of stromal staining			
Intensity grade	Weak		Strong		Weak		Strong	
Tu stage	0	1	2	3	0	1	2	3
Tu2	0	0	3	3	0	2	3	1
Tu3	1	3	3	7	0	4	5	5
Tu4	2	13	13	9	0	16	16	5
Total	19 (33%)		38 (67%)		22 (39%)		35 (61%)	
Node(−) neg.	2	3	6	5	0	6	5	5
Total	5 (9%)		11 (19%)		6 (11%)		10 (18%)	
Node(+) pos.	1	13	13	14	0	16	19	6
Total	14 (25%)		27 (47%)		16 (28%)		25 (44%)

Our data revealed an obvious correlation between immunoreactivity for MMP-13 and tumour progression only for strong staining, which decreased, and for minimal staining, which increased (Figure 4). Overall, the expression of MMP-13 declined with the progression of OSSC. The existing data regarding the diagnostic/prognostic value of MMP-13 are conflicting. Whilst Brinckerhoff et al. [52] have reported the existence of a positive correlation between the expression of MMP-13 and lymph-node involvement, Gottschlich et al. [53] have observed no such relationship. As compared with the other three MMPs investigated by us, strong staining for MMP-13 was observed in a higher proportion of patients with lymph-node involvement than in those without it. However, for the other MMPs investigated, although lymph-node involvement was associated with a high incidence of strong staining, no correlation existed between lymph-node involvement, strong immunostaining and the stage of the tumour.

### MMPs and OSCC invasion

As a next step in our study, the invasion front of each OSCC was evaluated histologically according to Bryne's malignancy-grading system. Four parameters were assessed: (1) degree of keratinization; (2) nuclear polymorphism; (3) tumour structure (pattern of invasion); and (4) host's cellular inflammatory response. These findings were then correlated with the immunostaining data for each MMP.

#### Degree of keratinization

**Figure 5.**
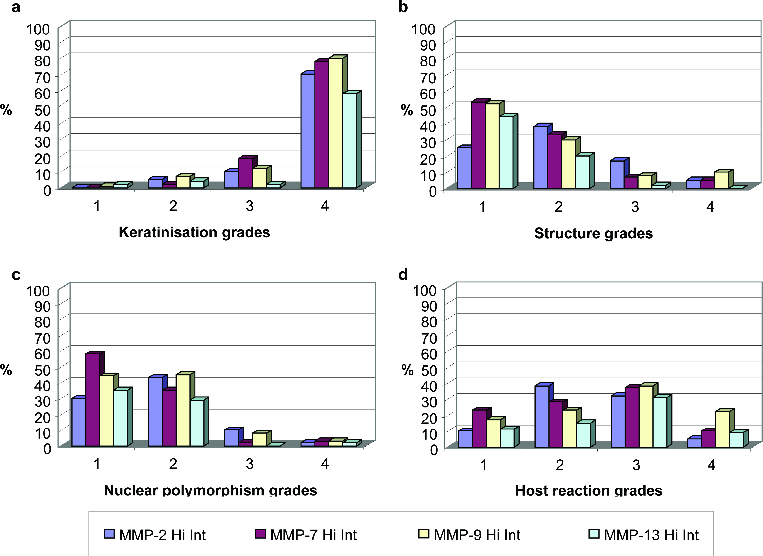
Distribution of MMP expression intensity (%) according to keratinization grades (a), structure grades (b), nuclear polymorphism grades (c) and host reaction grades (d).

Between 80% and 87% of the samples were given a grade of 4, i.e. no keratinization (0%–5% of the cells). In 70%–80% of these grade 4 cases, intratumoural staining for MMP-2, MMP-7, MMP-9 and MMP-13 was strong (Figure 5(a)). Highly keratinized regions (grade 1) did not stain strongly for any of these MMPs.

#### Structure (pattern of invasion)

According to the pattern of invasion, between 75% and 85% of the samples were given a grade of 1 (advancing) or 2 (infiltrating solid cords, etc.). In 75%–85% of these grade 1 and grade 2 cases, intratumoural staining for MMP-2, MMP-7, MMP-9 and MMP-13 was strong (Figure 5(b)). Generally, the presence of single or small groups of tumour cells within the stroma is a sign of high malignancy and poor prognosis.[4] In our experiments, this structural feature (grade 4 in Figure 5(b)) was associated with weak staining for MMP-7 and MMP-13.

#### Nuclear polymorphism

With regard to nuclear polymorphism, between 85% and 95% of the samples were given a grade of 1 (minimal) or 2 (moderate). In 80%–90% of these grade 1 and grade 2 cases, intratumoural staining for MMP-2, NNP-7, MMP-9 and MMP-13 was strong (Figure 5(c)). An extremely high degree of nuclear polymorphism (mature cells: 0%–25%), which occurred within regions where no BMs were evident, was likewise associated with minimal immunostaining for MMP-7 and MMP-13 (grade 4 in Figure 5(c)). Thus, MMP-7 and MMP-13 appeared to be absent from areas of diffuse invasion and high malignancy.

#### Host's cellular inflammatory response

The host's cellular immune response to a tumour reflects its potency. In our study, however, no consistent trend in this parameter was observed for any of the tested MMPs (Figure 5(d)). Grades 1 (marked), 2 (moderate) and 4 (none) were each observed in 15%–20% of the samples, and grade 3 (slight), in 37%. Weak intratumoural staining for MMP-2, MMP-7, MMP-9 and MMP-13 was not associated with any of the grades of the host's cellular inflammatory response.

### Final remarks

Our results that MMP-7 and MMP-13 were absent from areas of diffuse invasion and high malignancy, in good correlation with Bryne's malignancy grading of tumour structure and nuclear polymorphism, suggest that together these two MMPs might be useful as predictive markers of invasiveness in OSCC. This observation might possibly be considered to be associated with the mechanisms of action of these MMPs. MMP-7 cleaves plasminogen to angiostatin and type-XVIII collagen to endostatin – events which lead to an inhibition of angiogenesis and possibly to a retardation of tumour growth.[49] It could be speculated that an increase in the substrate activity of MMP-7 possibly accounts for the overall decrease in the expression of this enzyme with an advance in the stage of the tumour. As the malignancy potential of the tumour increases, the ability of MMP-7 to retard its invasion declines considerably. The marked decline in the expression of MMP-13 that was associated with advanced stages of tumour invasion could reflect the fact that the BM has already been digested.

Together with TNM-staging of the patient, immunohistochemistry for MMP-7 and MMP-13 could facilitate diagnosis and an assessment of the prognosis. On the other hand, the fairly constant levels of expression of MMP-2 and MMP-9 during the evolution of OSCCs, and the lack of correlation between their patterns of immunostaining and Byrne's malignancy grading of either tumour structure or nuclear polymorphism, indicate that they are not likely to be considered suitable markers for the invasion potential of OSCCs. We believe that testing for MMPs in patients with OSCC could help to define the roles of MMPs in oncogenesis, ultimately leading to targeted therapy and an improvement in outcome.

## Conclusions

Our data revealed MMP-7 and MMP-13 to be down-regulated in highly invasive and malignant areas of OSCCs. Their patterns of immunostaining, unlike those of MMP-2 and MMP-9, are very likely to afford an indication of the invasion potential of the tumour. Our findings indicate that MMP-7 and MMP-13 are reliable markers of the invasion potential of OSCC, whereas MMPs 2 and 9 are not. The patterns of immunostaining of MMP-7 and MMP-13 correlated well with Bryne's malignancy grading of tumour structure and nuclear polymorphism, thereby indicating that these MMPs can be relevant in gauging the malignancy potential of OSCC. Together with TNM-staging of the patient, immunohistochemistry for MMP-7 and MMP-13 could facilitate diagnosis and an assessment of the prognosis. We believe that testing for MMPs in patients with OSCC could help to define their roles in oncogenesis, ultimately leading to targeted therapy and an improvement in outcome.
